# Positive selection over the mitochondrial genome and its role in the diversification of gentoo penguins in response to adaptation in isolation

**DOI:** 10.1038/s41598-022-07562-0

**Published:** 2022-03-08

**Authors:** D. Noll, F. Leon, D. Brandt, P. Pistorius, C. Le Bohec, F. Bonadonna, P. N. Trathan, A. Barbosa, A. Raya Rey, G. P. M. Dantas, R. C. K. Bowie, E. Poulin, J. A. Vianna

**Affiliations:** 1 Departamento de Ecosistemas y Medio Ambiente, Pontificia Universidad Católica de Chile, Vicuña Mackenna 4860, Macul, Santiago, Chile; 2 Millennium Institute Biodiversity of Antarctic and Subantarctic Ecosystems (BASE), Santiago, Chile; 3 Facultad de Ciencias, Instituto de Ecología y Biodiversidad, Universidad de Chile, Santiago, Chile; 4 Department of Integrative Biology, University of California, 3101 Valley Life Science Building, Berkeley, CA 94720 USA; 5 Department of Zoology, 11DST/NRF Centre of Excellence at the Percy FitzPatrick Institute for African Ornithology, Nelson Mandela University, Port Elizabeth, South Africa; 6 CNRS, IPHC UMR 7178, Université de Strasbourg, 67000 Strasbourg, France; 7 Département de Biologie Polaire, Centre Scientifique de Monaco, 98000 Monaco City, Monaco; 8 CEFE UMR 5175, CNRS, Université de Montpellier, Université Paul-Valéry Montpellier, EPHE, Montpellier Cedex 5, France; 9 British Antarctic Survey, Cambridge, UK; 10 Departamento de Ecología Evolutiva, Museo Nacional de Ciencias Naturales, CSIC, Madrid, Spain; 11 Centro Austral de Investigaciones Científicas – Consejo Nacional de Investigaciones Científicas y Técnicas (CADIC-CONICET), Ushuaia, Argentina; 12 Instituto de Ciencias Polares, Ambiente y Recursos Naturales, Universidad Nacional de Tierra del Fuego, Ushuaia, Argentina; 13 Wildlife Conservation Society, Buenos Aires, Argentina; 14 PPG in Vertebrate Biology, Pontificia Universidade Católica de Minas Gerais, Belo Horizonte, Brazil; 15 Museum of Vertebrate Zoology and Department of Integrative Biology, University of California, 3101 Valley Life Science Building, Berkeley, CA 94720 USA; 16 Fondo de Desarrollo de Áreas Prioritarias (FONDAP), Center for Genome Regulation (CRG), Santiago, Chile

**Keywords:** Evolution, Evolutionary genetics

## Abstract

Although mitochondrial DNA has been widely used in phylogeography, evidence has emerged that factors such as climate, food availability, and environmental pressures that produce high levels of stress can exert a strong influence on mitochondrial genomes, to the point of promoting the persistence of certain genotypes in order to compensate for the metabolic requirements of the local environment. As recently discovered, the gentoo penguins (*Pygoscelis papua*) comprise four highly divergent lineages across their distribution spanning the Antarctic and sub-Antarctic regions. Gentoo penguins therefore represent a suitable animal model to study adaptive processes across divergent environments. Based on 62 mitogenomes that we obtained from nine locations spanning all four gentoo penguin lineages, we demonstrated lineage-specific nucleotide substitutions for various genes, but only lineage-specific amino acid replacements for the ND1 and ND5 protein-coding genes. Purifying selection (dN/dS < 1) is the main driving force in the protein-coding genes that shape the diversity of mitogenomes in gentoo penguins. Positive selection (dN/dS > 1) was mostly present in codons of the Complex I (NADH genes), supported by two different codon-based methods at the ND1 and ND4 in the most divergent lineages, the eastern gentoo penguin from Crozet and Marion Islands and the southern gentoo penguin from Antarctica respectively. Additionally, ND5 and ATP6 were under selection in the branches of the phylogeny involving all gentoo penguins except the eastern lineage. Our study suggests that local adaptation of gentoo penguins has emerged as a response to environmental variability promoting the fixation of mitochondrial haplotypes in a non-random manner. Mitogenome adaptation is thus likely to have been associated with gentoo penguin diversification across the Southern Ocean and to have promoted their survival in extreme environments such as Antarctica. Such selective processes on the mitochondrial genome may also be responsible for the discordance detected between nuclear- and mitochondrial-based phylogenies of gentoo penguin lineages.

## Introduction

Identifying the microevolutionary processes that underlie diversification of species is key to understanding the current distribution of genetic diversity and the possible future response of the biota to climate change. Natural selection should favor certain phenotypes and genotypes in a particular environment, leading to local adaptation^1^ and, consequently, promote population divergence and over time speciation.

Although mitochondrial DNA has traditionally been used as a neutral marker in phylogeography, evidence has emerged that supports the idea that the environment can act as a selective force promoting haplotype variation and potentially altering mitochondrial function and heat production^2–4^. Mitochondria provide the chemical energy necessary for the maintenance of cellular processes through the oxidative phosphorylation pathway (OXPHOS), which operates in the mitochondrial inner membrane^5^, producing cellular ATP, heat, and reactive oxygen species (ROS). The OXPHOS system depends on the interaction of five protein complexes, which are composed of subunits encoded by 13 mitochondrial protein-coding genes and ~ 80 nuclear-encoded genes (N-mt)^6^. Complexes I, III, and IV make up the respirasome, which drives proton-translocation to the intermembrane space, liberating energy (ATP) during electron transfer from NADH to O_2_ and thereby establishing a proton gradient across the inner mitochondrial membrane^7,8^. Complex V (ATP synthase) uses this proton-motive force to drive ATP synthesis. The nuclear-encoded proteins serve as electron carriers, alternative electron inputs, and assembly factors^6^, requiring close compatibility between mt and N-mt protein subunits. This is enabled through a strong selective pressure for N-mt compatibility and optimal performance that maintains a mito-nuclear interaction in the OXPHOS system^9,10^.

Different processes drive how selection acts on mitochondrial genes that may alter mitochondrial function. Organisms that inhabit environments differentiated that comprise different climates or different food availability regimes will have different metabolic requirements. These requirements favor nucleotide substitutions that produce amino acid replacements in the protein subunits encoded in mtDNA, allowing organisms to adapt to their local environment^11–14^. Organisms endotherms that inhabit areas with low temperatures (*i.e.,* polar environments) will need to be efficient at producing heat, while organisms from regions with low food availability will require greater efficiency in the production of ATP^15^. Further, prolonged exposure to external stressors, like pathogenic agents, activates an immune response^16^ and the ROS synthesis pathways by genes of the mitochondrial Complex I^17,18^ resulting in high levels of oxidative stress^19^. This may also reflect a greater predominance of certain alleles over others, promoting a non-random distribution of genetic diversity in locally adapted populations.

Gentoo penguins (*Pygoscelis papua*) are widely distributed throughout the Southern Ocean. They mainly breed on sub-Antarctic Islands but are also found abundantly in the Antarctic Peninsula and maritime Antarctica^20^. Although they are treated as a single species, their taxonomy is still under review with recent studies having revealed the existence of at least four cryptic lineages: northern gentoo (from South America and Falkland/Malvinas Is.), southern gentoo (Antarctica and maritime Antarctica), eastern gentoo (Crozet, Marion, and possibly Macquarie Is., to the north of the Antarctic Polar Front: AFP), and southeastern gentoo (from Kerguelen Is.)^21–24^. Divergence times have been estimated in past studies^21–24^, where analyses of ultraconserved elements (UCEs) distributed across the genome placed the split of the gentoo penguin lineages during the Pleistocene, between 0.47 and 1.26 Mya^23^ in response to the extension and retreat of the ice during this period which has been described as the main precursor of diversification of the marine fauna of the Southern Ocean^25^. Unlike most penguin species, gentoo penguins are inshore foragers, generally remaining resident year-round, with a generalist diet that is dependent on prey availability in their local environment^26–28^. Therefore, genetic drift could explain, in part, the high levels of genetic differentiation among gentoo lineages. The existence of several independent evolutionary lineages, with virtually no admixing (gene flow) across the Southern Ocean, is conductive for local adaptive processes to occur. This is particularly relevant when considering the wide range of environmental conditions to which different lineages are subjected, imposed by oceanographic barriers (oceanic fronts) that are important in structuring of penguin populations^23,29–33^. In the case of gentoo penguins, these environmental differences have been characterized, which highlighted important differences in the marine environment close to their breeding colonies^21^. In this sense, Vianna et al.^23^ suggested that the divergence of the four lineages of gentoo penguins occurs across thermal and salinity gradients. Additionally, using niche modeling, Pertierra et al.^21^ revealed that the colonies belonging to the eastern lineage, located north of the AFP, experience a very limited range of climatic variability in areas with low primary productivity marine environment. In contrast to the eastern lineage, the northern lineage occupies an environment with elevated levels of primary productivity; the southern lineage is strongly influenced by the formation of sea ice and seasonal melting that affects sea surface temperature and salinity, which in turn drives the absence (winter) or proliferation (summer) of primary productivity. Although the southeastern lineage differs in characteristics of the terrestrial and marine environments, Pertierra et al.^21^ report a signal of niche overlap (6-7%) among this lineage with the northern and southern, but it is too small to homogenize the environments, suggesting ecological segregation. Therefore, it is possible that the divergence of the gentoo penguin lineages has been promoted local adaptive processes that were driven by the different environment induced metabolic requirements of each lineage.

To evaluate whether the diversification of gentoo penguins was accompanied by natural selection acting on mitochondrial function, we analyzed the mitogenomes of 62 gentoo penguins encompassing all four evolutionary lineages: eastern, southeastern, northern, and southern gentoos. We evaluated the divergence patterns in mitochondrial coding sequences and used different approaches to detect selection signals on mitogenomes and mutations that lead to functional diversification. This mitogenomic information will provide an important guide for understanding the evolutionary consequences and adaptive mechanisms of widely distributed organisms in heterogeneous environments of the Southern Ocean.

## Results

### Sequences variation among gentoo penguin mitogenomes

**Figure 1:**
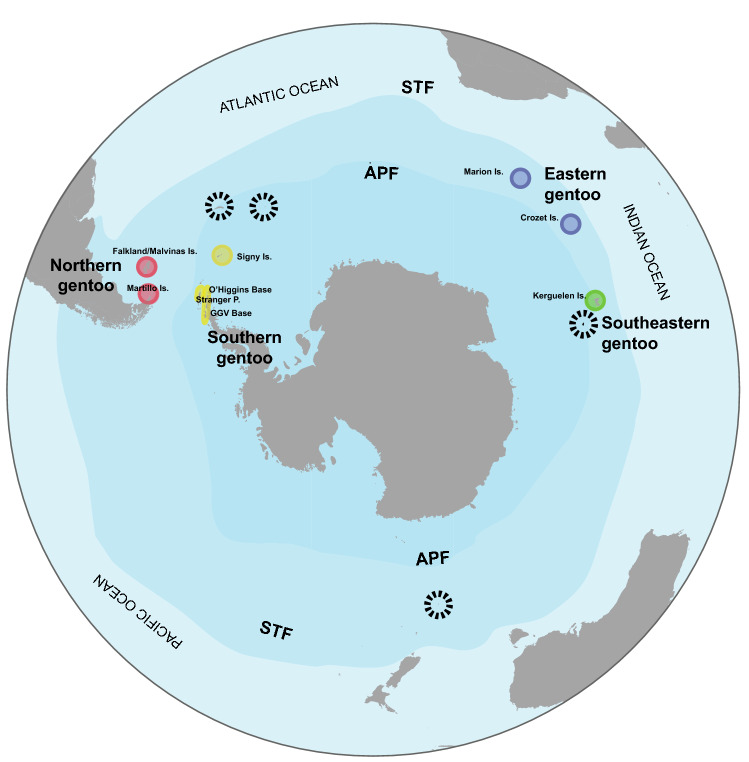
Distribution of gentoo penguins around the Southern Ocean. The figure shows the sample locations (colored circles): northern gentoo: Martillo and Falkland/Malvinas I.; southern gentoo: Signy Island, O’Higgins base, Stranger Point and Gabriel Gonzalez Videla base; southeastern gentoo: Courbet Peninsula, Kerguelen I.; eastern gentoo: Marion and Crozet I. and open circles represent areas with unsampled colonies. Map images provided by Shutterstock database and edited in Adobe Illustrator.

**Figure 2:**
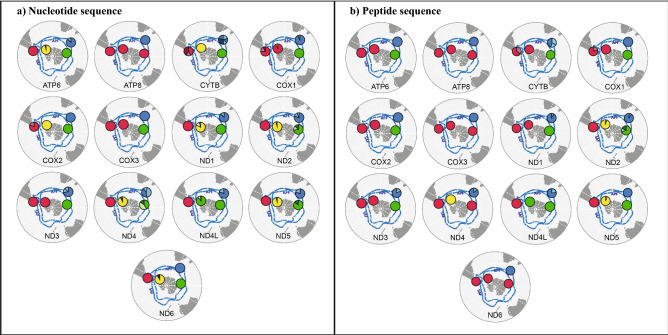
(**a**) Haplotype distribution of gentoo penguin lineages around the Southern Ocean; (**b**) Distribution of haplotypes translated into amino acid sequences. Map images provided by Shutterstock database and edited in Adobe Illustrator.

A total of 62 gentoo penguin individuals from nine breeding locations were analyzed (Fig. 1, Table S1). Recovered mitogenome sequences presented slight variations in their length (16,993–16,998 bp) attributed mainly to differences in the control region (Table S2). Gentoo penguin mitogenomes contained the 37 genes usually found in bird mitochondrial genomes^34^ and rearrangements of genes and genomic regions were not observed since the same reference genome was used for all lineages. Very low levels of nucleotide diversity were found in all protein coding genes (Table 1), with ATP8 and COX3 having the lowest values (0.0046 and 0.0045 respectively). This result, together with the low number of haplotypes, suggests that these genes are highly conserved among lineages, and likely subject to purifying selection. On the other hand, ATP6, CYTB, COX2, ND1, ND2, ND4, ND5 and ND6 contained haplotypes that are unique to each of the gentoo penguin lineages (Fig. 2a, S1). We found that ND2, ND4 and ND5 produced exclusive peptides for each lineage, while the different haplotypes detected in the other genes comprised synonymous substitutions that, in most cases, produced the same peptide (Fig. 2b). For all genes, the haplotypes from the eastern lineage were always the most differentiated, even within the most conserved loci such as ATP8 and COX3 (Fig. S1). In these two genes, by translating the sequences into amino acids, it was possible to identify exclusive peptides of the eastern lineage for all protein coding genes (Fig. 2b), including the most conserved, ATP8 (Fig. S1). With respect to ribosomal genes, we identified five haplotypes for 12S gene, where the eastern and southeastern lineages were differentiated (Fig. S2a). In the case of the 16S gene, we recovered unique haplotypes for each lineage, which also showed greater differentiation of the eastern and southeastern lineages, with 13 and 5 mutational steps, respectively (Fig. S2b).

**Table 1:** Indices of diversity of mt-genes (N: number of sequences, H: number of haplotypes, S: number of polymorphic sites, π: nucleotide diversity, Np: number of peptides generated after translation of sequences, ω: dN/dS).

mt-gen	N	Size (bp)	H	S	π	Np	ω
ATP6	62	681	6	28	0.0127	3	0.038
ATP8	62	162	2	2	0.0046	2	–
CYTB	62	1137	10	30	0.0088	6	0.100
COX1	62	1533	7	33	0.0076	4	0.019
COX2	62	675	5	16	0.0084	3	0.021
COX3	62	783	3	11	0.0045	2	0.021
ND1	62	978	7	18	0.0065	4	0.075
ND2	62	1029	9	22	0.0071	8	0.179
ND3	62	348	5	11	0.0105	4	0.142
ND4	62	1368	10	35	0.0086	4	0.110
ND4L	62	294	6	7	0.0055	4	0.248
ND5	62	1803	7	41	0.0080	5	0.097
ND6	62	516	7	18	0.0109	2	0.048

*ω was not estimated for ATP8 because it only has 2 haplotypes.

We detected different levels of genetic divergence among the recovered mitogenomes (Fig. S3, Table S3). The lowest differentiation was between northern and southern gentoos (0.0022–0.0029), while eastern gentoos showed the highest levels of genetic differentiation (0.0223–0.0234 eastern/southeastern gentoos, 0.0209–0.0218 eastern/northern gentoo and 0.0211–0.0220 eastern/southern gentoo). Intermediate levels of divergence were observed among southeastern with northern and southern gentoos, where the values of genetic distance were much lower than those detected for the eastern lineage (0.0077–0.0084 southeastern/northern and 0.0074–0.0083 southeastern/southern gentoo), despite the greater geographical distance.

### Phylogenetic reconstruction

**Figure 3:**
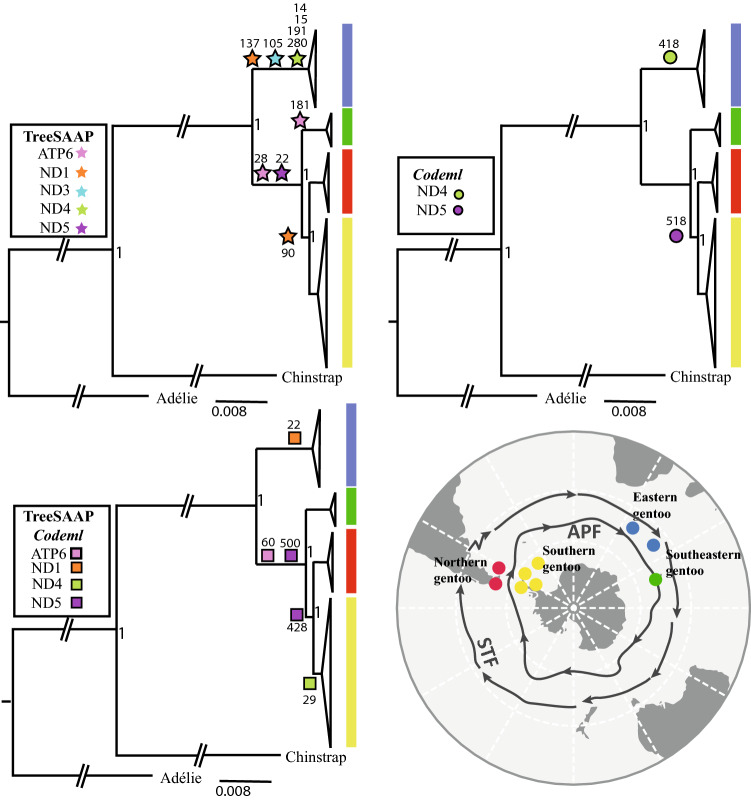
Bayesian phylogenetic reconstruction obtained from partitions of 13 protein coding genes (11,170 bp). On the phylogenies, stars, circles, and squares represent codons under selection obtained using different approaches. Stars and circles represent codons with positive selection signals obtained from only TreeSAAP or only codeml analyses, respectively. Squares represent sites under selection using both programs. Abbreviations on the map: APF (Antarctic Polar Front) and STF (Sub-Tropical Front). All nodes were supported by PP = 1.0. Map images provided by Shutterstock database and edited in Adobe Illustrator.

**Figure 4:**
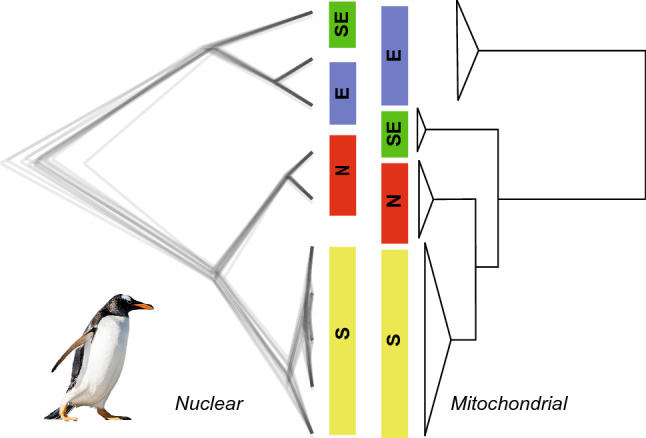
Mito-nuclear discordance between topologies of gentoo penguins. Left: Genomic phylogeny (SNAPP) generated using 4429 SNPs in Pertierra et al. (2020). Right: Bayesian phylogeny from 13 mitochondrial protein coding genes (11,170 bp).

The concatenated protein coding genes resulted in a sequence of 11,170 bp. Since the sites within a codon can evolve at different rates, a partition scheme was established considering the position of the nucleotide within the codon. In this way, the best partition scheme produced 15 sub-sets, where its corresponding nucleotide substitution model was estimated (Table S4). Those positions within genes that presented the same evolutionary pattern were grouped within the same set. The partition scheme was considered for the reconstruction of the Bayesian phylogeny, in which the monophyly of each lineage was recovered (Fig. 3) with high node support (Potential scale reduction factor (PSRF) = 1000; Effective Sample Size (ESS) = 8022). The phylogenetic reconstruction generated from the mitochondrial partitions recovered the same topology as those generated from the control region in previous studies^21,24^. It thus supported the finding that the eastern gentoo penguin clade was the first to diverge from the remaining clades, followed by the southeastern lineage as a sister clade of the southern and northern clade. However, the topology generated with mitochondrial markers differs from the reconstructions obtained by previous studies using nuclear DNA markers^21^, which places the eastern lineage as a sister clade to the southeastern lineage, and the other two lineages as sister groups on a separate branch (Fig. 4).

### Evidence of selection signals in mitochondrial genes

We implemented different methods to identify putative codons under selection in gentoo penguin mitochondrial genes. First, we used a codon-based approach to estimate the ratio of non-synonymous (dN) to synonymous (dS) nucleotide substitutions (ω = dN/dS) to detect putative codons under selection. FUBAR detects codons with signal of purifying or positive selection evaluating the posterior probability of each codon belonging to each class of ω, while *codeml* is a likelihood approach that suggests genes under positive selection through the contrast of a neutral model of evolution with a positive selection model. As expected, we found a large number of codons with a signal of purifying selection in all protein coding genes, but particularly ATP6, COX1 and ND6 (Table S5). Codons under positive selection were less prevalent than purifying selection and were principally present genes of Complex I of the mitochondrion. FUBAR identified two codons under positive selection in ND2 and one codon in ND5 (Table 2). The same genes showed a signal of selection when a *codeml* site-model was used, identifying an additional codon in ND5 (Tables 2 and 3), suggesting that 0.7% of the sites in ND2 and 0.5% of the sites in ND5 have evolved under positive selection (M2a, Table S6). With respect to ND1, Bayes Empirical Bayes (BEB) detected a signal of selection in one codon (Table 2), but this gene was not significant when estimating Δ LRT (Table 3).

**Table 2:** Detection of positive selection in codons of OXPHOS mitochondrial genes using four methods: TreeSAAP, Fubar and *codeml*.

OXPHOS Complex	Gene	Codon	TreeSAAP				*codeml* BS		*codeml* SM		Fubar
			From–to	Branch	Cat	Prop	Branch	PP	M2	M8	
V	ATP6	28	Ala–Thr	SE + N + S	6	↓Pα	ns	ns	ns	ns	ns
		60	Thr–Ala	SE + N + S	6	↑Pα	ns	ns	ns	0.75*	ns
		181	Ala–Thr	SE	6	↓Pα	ns	ns	ns	ns	ns
I	ND1	22	Ile–Val	E	8	↑EC	SE + N + S	0.75*	ns	ns	ns
		90	Ile–Leu	N + S	8	↑EC	ns	ns	ns	ns	ns
		137	Ile–Val	E	8	↑EC	ns	ns	ns	ns	ns
I	ND2	2		ns	ns	ns	ns	ns	1***	1***	0.921
		321		ns	ns	ns	ns	ns	1***	1***	0.929
I	ND3	105	Tyr–His	E	7	↑αm	ns	ns	ns	ns	ns
I	ND4	14	Thr–Ala	E	6	↑ Pα	ns	ns	ns	ns	ns
		15	Ala–Thr	E	6	↓ Pα	ns	ns	ns	ns	ns
		29	Thr–Ala	S	6	↑ Pα	N + S	0.78*	ns	ns	ns
		191	Thr–Ala	E	6	↑ Pα	ns	ns	ns	ns	ns
		280	Thr–Ala	E	6	↑ Pα	ns	ns	ns	ns	ns
		418		ns	ns	ns	ns	ns	0.78*	0.81**	ns
I	ND5	22	Thr–Ala	SE + N + S	6	↑Pα	ns	ns	ns	ns	ns
		428	Thr–Ala	N + S	6	↑Pα	ns	ns	0.89**	0.89**	0.92
		500	Ala–Thr	SE + N + S	6	↓Pα	ns	ns	0.79*	0.81**	ns
		518		ns	ns	ns	N + S	0.96**	ns	ns	ns

Pα: α-helical tendencies; αm: Power to be at the middle of alpha-helix; *EC* equilibrium constant, *E* eastern gentoo, *SE* southeastern gentoo, *N* northern, *S* southern gentoo. *Codeml* BS (Branch-site model) and SM (site model).

Posterior probability estimates by Bayes Empirical Bayes in site and branch-site models (PP: Posterior probability; *:PP > 0.7, **:PP > 0.8, ***: PP > 0.99).

**Table 3:** Estimation ΔLRT of M1a/M2a and M7/M8 nested *codeml* site models (*p < 0.05) to detect genes candidates of positive selection.

Gen	Alternative model	Null model	ΔLRT	DF	*p*-value
ATP6	Model 2a	Model 1a	0.0150	2	0.9920
ATP6	Model 8	Model 7	0.5650	2	0.7530
COX1	Model 2a	Model 1a	0.0002	2	0.9998
COX1	Model 8	Model 7	0	2	1
COX2	Model 2a	Model 1a	0.0001	2	0.9999
COX2	Model 8	Model 7	0	2	1
COX3	Model 2a	Model 1a	0	2	1
COX3	Model 8	Model 7	0	2	1
CYTB	Model 2a	Model 1a	0.0002	2	0.9990
CYTB	Model 8	Model 7	0	2	1
ND1	Model 2a	Model 1a	0	2	1
ND1	Model 8	Model 7	0	2	1
ND2	Model 2a	Model 1a	12.0300	2	0.0024*
ND2	Model 8	Model 7	12.4700	2	0.0020*
ND3	Model 2a	Model 1a	0	2	1
ND3	Model 8	Model 7	0	2	1
ND4	Model 2a	Model 1a	0.3390	2	0.8430
ND4	Model 8	Model 7	2.1970	2	0.3330
ND4L	Model 2a	Model 1a	0.0001	2	0.9990
ND4L	Model 8	Model 7	0.0053	2	0.9990
ND5	Model 2a	Model 1a	2.1680	2	0.3380
ND5	Model 8	Model 7	9.4800	2	0.0088*
ND6	Model 2a	Model 1a	0	2	1
ND6	Model 8	Model 7	0	2	1

To assess the existence of selection within a particular gentoo penguin lineage, each branch of the phylogeny was independently tested. All combinations showed strong evidence of purifying selection acting on mitochondrial protein coding genes (Table S7). Branch-site model *A* detected codons with evidence of positive selection in ND5 in the southeastern lineage (Table 4). When we evaluated the spatial distribution of peptides (Fig. 2), we observed sequences shared between lineages in most genes mainly between northern, southern, and southeastern gentoos, and the eastern lineage always had a different sequence. For this reason, we grouped the most related lineages to look for evidence of selection: (1) N + S and (2) N + S + SE gentoos. We found signals of positive selection in ATP6 when we tested the N + S + SE lineages, while in the N + S branches, selection was detected in ND5 (Table 4, Fig. 3). ND2 showed high levels of significance when evaluating selection with site models, but it was not detected at the branch level. In the #2ND2 (detected using site model), the eastern, southeastern, and southern lineages have the amino acid asparagine except for one individual from the southern lineage, which is the only individual that shares the amino acid serine with the northern lineage. This substitution could be a false positive, or it could reflect incomplete lineage sorting between sibling lineages.

**Table 4:** Estimation ΔLRT of nested *codeml* branch-site model A (*p < 0.05).

Branch	Gen	ΔLRT (Null)	ΔLRT (M1a)	df	*p*-value (null)	*p*-value (M1a)
SE + N + S	ATP6	0.50	3.98	1	0.478	0.046*
SE + N + S	ND1	2.55	2.01	1	0.110	0.150
N + S	ND4	0.94	0.94	1	0.330	0.330
SE	ND5	7.94	0.01	1	0.005*	0.931
N + S	ND5	8.65	2.72	1	0.003*	0.099

We used the program TreeSAAP to evaluate if the amino acid substitutions generate a radical change in the physicochemical properties. Although in all genes a larger proportion of conservative amino acid changes were observed relative to radical changes, twelve sites in genes of Complex I, and three sites in ATP6 were identified as being under positive selection using TreeSAAP (Table 2, Fig. 3). This suggests the presence of amino acid replacement with different physicochemical properties. Signals of selection were recovered in the codon #137ND1 located in the conserved domain, which contains the ubiquinone binding site, a region that regulates the reduction of ubiquinone and the translocation of protons. Specifically, the eastern lineage presented an amino acid valine while all individuals from the other lineages exhibit isoleucine, a substitution categorized as a change in the constant equilibrium of ionization property. Signals of selection detected using at least two methods (TreeSAAP and one based on dN/dS) suggest selection in #22ND1, #29ND4 located in the N-terminal domain, and in the codons 428 and 500 of the C-terminal region of the ND5 gene (Table 2, Fig. 3). In addition, both methods detected a signal of selection at #60ATP6, a nucleotide substitution placed on the cytoplasmic domain of the subunit.

## Discussion

We examined the divergence patterns of gentoo penguin mitogenomes around the Southern Ocean and evaluated the potential role of natural selection acting in different environments on mitochondrial DNA evolution. Gentoo penguins are broadly distributed around the Southern Ocean and show strong population genetic structuring due to their high residency patterns and foraging behavior close to the coast^35–38^. The levels of mitochondrial divergence observed are higher than those previously described in rockhopper penguins *Eudyptes chrysocome, E. filholi* and *E. moseleyi*^32^ and the two putative species of little penguins *Eudyptula minor* and *Eudyptula novaehollandiae*^39,40^. In other penguin species, such as chinstrap *P. antarcticus*^41,42^, macaroni/royal *E. chrysolophus* and *E. schlegeli*^32^ and king penguin *Aptenodytes patagonicus*^43^ very limited or no population structure is evident throughout their geographical distributions. Despite geographic isolation among gentoo lineages and the fact that mtDNA is inherited as a linked unit, we identified different patterns of genetic diversity among protein coding genes, suggesting that mitochondrial proteins are subject to different degrees of selection (evolutionary dynamics)^44^.

Species that inhabit high latitude environments with extreme climates such as the sub-Antarctic and Antarctic regions could be subject to unique selective pressures related to high energy demands^45^. Stressors associated with thermoregulation and seasonal food availability could lead to metabolic changes based on mitochondrial energy regulation mechanisms^46–48^. Mitochondrial function plays a leading role in the regulation of energy metabolism, and positive selection on mitochondrial protein coding genes can drive population divergence and speciation^49^.

We detected potentially relevant fixed amino acid differences across different mitochondrial genes and amino acid variations in functionally important regions of these genes. Such differences could have functional implications that can lead to local adaptation^13^. Although the effects of genetic drift should not be disregarded, our results suggest a non-random geographical pattern of selection acting on mitochondrial genes. Different levels of selective pressure appeared to act principally on genes of Complex I and on one gene of the Complex V, leading to changes the physicochemical properties of some amino acids and non-synonymous substitutions in the eastern, southern, and southeastern + northern + southern branches of the phylogeny (Fig. 3). Complex I (NADH: ubiquinone oxidoreductase) and Complex V (ATP synthase) play a central role in cellular energy production^50^ and are the mitochondrial complexes that present the greatest evidence of selection in marine organisms^51–55^.

The relationship between mitochondrial genes under positive selection and climatic heterogeneity has been widely studied. In polar environments, signals of positive selection on mitochondrial genes could be associated with higher aerobic capacity to have a more efficient metabolism relative to the generation of heat in endothermic organisms^4,15^. In this sense, Complex I genes are largely involved in adaptation to cold regions, as observed in the Arctic environment, where polymorphisms under selection in ND4 in Atlantic salmon^52^ and ND5 in humans from Siberia^4^, are concordant with the ND4 and ND5 regions being under selection in southern gentoos (Fig. 3). In the case of sites from ND5, mutations placed in the piston arm of the protein have been observed to affect proton pumping, influencing fitness during the evolution of some species^54^, and consequently could improve ATP production and maintain aerobic scope in the cold. Surprisingly, we found evidence of positive selection acting only in genes of the eastern lineage, mainly in ND1 and ND4 genes, suggesting signals of sub-Antarctic adaptation. ND1 plays an essential role in the activity of Complex I, and mutations in the conserved domain can affect the ubiquinone-binding site^56^, and therefore the efficiency of proton translocation. Eastern gentoo penguin lineage showed signals of selection in the physicochemical properties of the protein at position #137ND1, which is located in the cytoplasmic domain that contains that binding site. Changes in this region may influence metabolic efficiency in different species, including fishes^52,57^ and mammals^58^ and could be an indicator of adaptive evolution. Codons under selection in ND4 (#191ND4 and #280ND4) in the eastern lineage are placed in the Mrp antiporter membrane subunit, where the reduction of ubiquinone and the translocation of protons are regulated^59,60^. This region was previously found to be under selection in penguins from contrasting environments, particularly from the Galápagos Islands and Antarctica^61^. In this case, a strong correlation between ND4 and sea surface temperature was detected. Southeastern, northern and southern lineages showed a selection signal in #60ATP6, a mutation placed on the cytoplasmic domain of the protein. This gene participates directly in the proton flow in the Complex V, generating a potential gradient with the phosphorylation of ADP^62^. In this sense, selected amino acid substitutions could alter the efficiency of ATP synthesis between southeastern/northern/southern and eastern gentoo penguin lineages.

Eastern gentoo penguins exhibited the greatest nucleotide and amino acid differentiation across all protein coding genes, even in the most conserved ones such as ATP8 and COX3. This contrasts with what is observed in southeastern, northern, and southern gentoos, which shared haplotypes, and where many of the substitutions tend to be synonymous (Fig. 2). A possible explanation can be attributed a current, the Antarctic Polar Front (AFP) separating these populations, but the environments associated with these gentoo penguins breeding localities on either side of the AFP not being sufficiently different to induce local adaption. In this way, it is possible that given the great environmental breadth among gentoo penguin lineages reported by Pertierra et al.^21^ in bioclimatic variables such as sea surface temperature, salinity and primary productivity, the observed mitochondrial genotypes allow for wide ranges of climatic tolerance of the individuals of these three lineages. Despite their current isolation, analyses of whole genomes have reported past introgression signals between the ancestral branch of northern/southern and the southeastern gentoos^23^, but with no sign of admixture at present^21,24,29,31^. In contrast, gentoos from the eastern lineage inhabit environments not only with reduced levels of primary productivity but also the greater amplitude of the maximum and minimum values in terms of salinity and sea surface temperature. This may have driven the observed signal of position selection, which is indicative of local adaptation that we detected.

Microevolutionary forces (drift, migration, mutation, and selection) play a central role in the differentiation of populations. Nuclear and mitochondrial genomes are subject to different tempos and modes of evolution^5^, and therefore, the observed patterns of divergence among gentoo lineages may be associated with the different modes of inheritance and functional specialization of the genomes. This can lead to incipient speciation through mito-nuclear functional compensation^63^. It has been suggested that species showing discordance in the divergence patterns between mtDNA and nDNA could be valuable models with which to investigate the relative roles of natural selection and neutrality acting over mitogenomes^64^. In gentoo penguins, mitochondrial DNA lineages are highly divergent consistent with results from genome-wide SNP analyses, the topologies of the resultant phylogenies generated from genomic data are discordant with the observed mitochondrial topologies (Fig. 4). Nuclear data group the eastern lineage as a sister clade to the southeastern lineage, and on another branch place the northern and southern gentoo as a sister group^21,23^. However, the topology obtained from mitochondrial information indicates that the eastern lineage is the first branching taxa to diverge from the remaining ones. This incongruence between mitochondrial and nuclear DNA is consistent with our results that selection has played a stronger role on the eastern lineage, which in a phylogenetic reconstruction would make it seem more divergent from other lineages than it really is.

Similar patterns of mito-nuclear discordance have been observed in birds^64–66^ and could occur as a consequence of different selection regimes, incomplete lineage sorting, hybridization/introgression processes^67^, or different demographic dynamics^68^. What microevolutionary forces are operating on gentoo penguin mitogenomes that promote discordance between nuclear and mitochondrial phylogenetic hypotheses? Aspects as asymmetric gene flow are unlikely because nuclear and mitochondrial markers suggest complete isolation of the lineages. Further, the pool of samples used in this study contains a random mix of males and females used to perform mitochondrial^24^ and nuclear^21^ phylogenetic reconstructions. The high degree of conservatism in nucleotide and amino acid sequences among northern, southern, and southeastern lineages, can be attributed to incomplete lineage sorting, but the effect of purifying selection acting on mitochondrial genes cannot be ignored, which would maintain a pool of genotypes to face metabolic requirements in these environments given the large amplitudes of interannual variability^21^. The mitochondrial control region was used in Vianna et al.^24^ to infer the demographic history, with a signal of population expansion detected only in the southern lineage. Therefore, it is possible to discard the effect of the historical demography acting over mitochondrial genes in the northern, eastern, and southeastern lineages and infer that the topology of the mitochondrial phylogeny would be influenced by strong positive selection pressure over the eastern lineage.

Some polymorphisms could be under co-evolution between nuclear and mitochondrial proteins due to environmental and intergenomic interactions^69^. In this context, there is strong evidence that mitochondrial protein coding genes play a central role in the regulation of several cellular activities, acting in conjunction with the innate immune response, particularly with respect to antibacterial immunity^16,70^. OXPHOS is the main cellular source of ROS and, under specific metabolic or stress conditions, the process augments mitochondrial superoxide generation^17,71^, resulting in high levels of oxidative stress within the cell^72^. There are different factors that can promote the generation of mitochondrial ROS, and they are not only associated with climatic variation. Toll-like receptors (TLRs) are a family of pattern-recognition receptors in the vertebrate immune system that are key to pathogen detection^73^. Signaling via TLRs directly augments mitochondrial ROS generation in macrophages in response to bacteria by coupling TLRs signaling to mitochondrial Complex I^16^. Within the range of the gentoo penguin, there are diverse abiotic (pollution, climatic conditions) and biotic (pathogens, parasites) factors that exhibit spatial variation^74–77^ and affect the prevalence and transmission of pathogens and immune response of gentoos^78,79^. In this sense, local selection on TLR4 and TLR5 alleles of gentoo penguins suggests pathogen-driven adaptation^22^. Environmental heterogeneity may be related to the differential prevalence of pathogens among geographic regions, favoring the predominance of certain alleles over others in response to the selective pressure of pathogens from the local environment. Studies on the diversity of parasites associated with the different gentoo penguin lineages and their relationship to selection patterns on genes involved in the immune response on each lineage are needed. The eastern lineage showed the lowest levels of diversity in TLR5^22^ and had a predominance of unique mitochondrial haplotypes (this study) with strong positive selection on conserved regions of genes. These attributes could be associated with high levels of mortality (32.6%) registered in this region over the last two decades^80^.

Results from this study highlights the importance of considering intraspecific diversity and cryptic speciation in biodiversity management and conservation. The case of the gentoo penguins clearly demonstrates this, since despite significant differences in terms of local adaptation and population dynamics that are indicative of four distinct evolutionary lineages, they are still managed as a single species.

## Methods

### Generation of mitogenome data set

We made use of samples from 62 individual Gentoo penguins previously obtained for other projects^21,24^, which covered a large part of the geographic distribution of gentoo penguins around the Southern Ocean (Fig. 1, Table S1). This sampling included the genomes of four gentoo penguins sequenced by Vianna et al.^23^ to complement the number of individuals sampled per location (Crozet Is, Kerguelen Is, Falkland/Malvinas Is and Antarctica). For this study, DNA was isolated from 58 blood samples using a salt extraction protocol^81^ with modifications^24^. We performed whole genome resequencing from 100 ng of genomic DNA, which was fragmented (~ 350 bp) to construct pair-end libraries using the Illumina TruSeq Nano kit. A total of six cycles of PCR were run for enrichment and resultant libraries were sequenced at 15 × coverage using an Illumina HiSeq X platform at MedGenome.

Read cleaning was performed using readCleaner (https://github.com/tplinderoth/ngsQC/), resulting in the removal of PCR duplicates, adapters, low quality bases, low complexity reads and potential contaminant reads of the raw data obtained from NGS. We then extract the mitochondrial reads from the cleaned fastq files with blatq (https://github.com/calacademy-research/BLATq) using a previously published gentoo penguin mitogenome as reference (GenBank access: NC_037702.1). Mitochondrial reads were assembled using Geneious Prime 2020.2 (https://www.geneious.com). Annotation was performed on the MITOS web server^82^ and visually corroborated by comparing with the composition of the reference. Additionally, we included the mitogenomes of Adélie (*P. adelie*, GenBank access: MK761002.1) and chinstrap (*P. antarcticus*, GenBank access: MK761001.1) penguins^61^ as outgroup and sister group for phylogenetic analysis.

Nucleotide sequences of coding regions were first aligned using MAFFT^83^. Nucleotide sequences were then translated into amino acids to determine if the haplotypes detected in each lineage produce proteins with different amino acid conformations. The sequences were translated into amino acids in Sequence Manipulation Suite SMS2^84^ and aligned again using MAFFT. Mindell et al.^85^ reports that some reptile and bird species have an extra nucleotide in the ND3 gene, which is not translated. In this sense, the nucleotide at position 174 of the ND3 gene was removed for the selection analysis to maintain the reading frame^61^. In the case of the ND6 gene, we worked with the reverse complement because it is encoded on the light strand.

We explored the patterns of nucleotide diversity among lineages in DNAsp v6.12.03^86^. To evaluate the genealogical relationship among haplotypes, we performed a Median Joining Network for each gene using PopArt (http://popart.otago.ac.nz). The degree of divergence among lineages was estimated through mitogenome pairwise distance using Mega X^87^.

### Detection of positive selection

The geographical distribution of haplotypes and protein sequences were evaluated for all 13 mtDNA protein coding genes; however, selection analyses were performed using 12 genes with the stop codons removed. It was not possible to evaluate selection signals for ATP8 due to the low number of haplotypes (two haplotypes). We use a codon-based approach to estimate the ratio of non-synonymous to synonymous nucleotide substitutions (dN/dS; ω) for each codon based on a phylogeny to detect selection signals among gentoo penguin lineages. Estimation of ω is used to detect selection signals and to measure the magnitude and direction of selection. When ω < 1, it suggests negative purifying selection, ω = 1 neutral evolution, whereas values of ω > 1 are indicative of positive selection^88^. The Fast, Unconstrained Bayesian AppRoximation (FUBAR)^89^ was used to detect codons under pervasive purifying or diversifying selection, evaluating the posterior probability (pp) of each codon belonging to each class of ω. In this case, codons with pp > 0.9 were assumed to be under selection. FUBAR is a package of the program HyPhy and was carried out on the Datamonkey platform (https://www.datamonkey.org/).

We also estimated ω through a maximum likelihood approach using the *codeml* package implemented in PAML^88^. Since *codeml* likelihood analysis is sensitive to the topology of the tree, we used a Bayesian phylogenetic reconstruction from the 13 protein coding mtDNA genes generates using MrBayes v.3.2.7^90^ via the CIPRES Science Gateway^91^. The analysis was performed using four replicate runs with four chains, 30 million generations, sampling every 1000 generations, and 25% of burnin. Fifteen partitions were defined by PartitionFinder 2.1.1^92^. The best scheme of partitions and the substitution models were obtained with Akaike Information Criterion (AICc) and a heuristic search was performed using the greedy algorithm^93,94^.

We ran a series of likelihood models to examine whether there was selection acting on particular codons independent of the phylogenetic branch. The site model assumes one ω ratio for all branches of the phylogeny and allows ω to vary among codons^95,96^: M1a (nearly neutral; two classes of ω ratios: ω_0_ < 1, ω_1_ = 1), M2a (positive selection; three site classes: ω_0_ < 1, ω_1_ = 1 and ω_2_ > 1), M7 (neutral model, ω varies according to the beta distribution), and M8 (positive selection; similar to M7 but with an additional codon class ω_S_ > 1). We used likelihood ratio test (LRT) to search for evidence of non-neutrality and evidence of positive selection comparing and evaluating the significance of two nested models (M1a vs. M2a and M7 vs. M8).

To evaluate if gentoo penguin lineages evolved under different selective pressures, branch-site model A^97^ was run in *codeml*. We tested all the branches and also grouped lineages that shared the same protein sequence (foreground: northern + southern (N + S) gentoo and northern + southern + southeastern (N + S + SE) gentoo). To test for the presence of positive selection in the foreground branch, we compared the alternative model to its respective null model (where ω_2_ is fixed assuming neutral evolution, i.e., ω_2_ = 1) and with the M1a (nearly neutral) model. For site and branch-site models, codons under positive selection were identified using the Bayes Empirical Bayes (BEB) method implemented in *Codeml*, and those who presented posterior probability (pp) > 0.7 were considered candidates for positive selection.

### Physicochemical changes in amino acids

Because adaptive change is reflected at the protein level, we used TreeSAAP to detect pronounced changes in physicochemical properties of amino acids^98^ on particular branches of the penguin phylogeny. This method compares the distribution of amino acid replacements and changes in physicochemical properties by assigning a value among 8 magnitude categories, where 1 is the most conservative, while 8 is the most radical. Only those genes that presented: (1) magnitude categories between 6 and 8; (2) amino acid changes with high support (*p* < 0.001); and (3) z-score above 3.09 were considered candidates of positive selection. In addition, we used the InterPro web server (https://www.ebi.ac.uk/interpro/) to determine the domain of the protein where the amino acid replacements occur.

## Supplementary Information

Supplementary Information.

## Data Availability

Gentoo penguins mitogenomes were deposited into GenBank (GenBank accession numbers: MZ571411-MZ571468). Penguin pictures were provided by the author Daly Noll.
