# Supplementary material for: Positive selection over the mitochondrial genome and its role in the diversification of gentoo penguins in response to adaptation in isolation
Source: Sci Rep. 2022 Mar 8;12:3767. doi: 10.1038/s41598-022-07562-0 (PMC8904570; doi:10.1038/s41598-022-07562-0)
Supplement: Supplementary file 1 — Supplementary Information. [file 41598_2022_7562_MOESM1_ESM.docx]

**Positive selection over the mitochondrial genome and its role in the diversification of gentoo penguins in response to adaptation in isolation**

Noll D.^1,2,3^, Leon, F.^1^, Brandt D.^4^, Pistorius P.^5^, Le Bohec, C. ^6,7^, Bonadonna, F.^8^, Trathan, P.N.^9^, Barbosa, A.^10^, Raya Rey A.^11,12,13^, Dantas G.P.M.^14^, Bowie R.C.K.^15^, Poulin E.^2,3^, Vianna J.A.^1,2,16*^

1- Pontificia Universidad Católica de Chile, Departamento de Ecosistemas y Medio Ambiente, Vicuña Mackenna 4860, Macul, Santiago, Chile.

2- Millennium Institute Biodiversity of Antarctic and Subantarctic Ecosystems (BASE), Santiago, Chile.

3- Instituto de Ecología y Biodiversidad, Facultad de Ciencias, Universidad de Chile, Santiago, Chile.

4- Department of Integrative Biology, 3101 Valley Life Science Building, University of California, Berkeley, CA 94720, USA.

5- 11DST/NRF Centre of Excellence at the Percy FitzPatrick Institute for African Ornithology, Department of Zoology, Nelson Mandela University, Port Elizabeth, South Africa.

6- CNRS, Université de Strasbourg, IPHC UMR 7178, F-67000, Strasbourg, France

7- Centre Scientifique de Monaco, Département de Biologie Polaire, MC 98000, Monaco City, Monaco

8- CEFE UMR 5175, CNRS, Université de Montpellier, Université Paul-Valéry Montpellier, EPHE, Montpellier Cedex 5, France.

9- British Antarctic Survey, Cambridge, UK.

10- Departamento de Ecología Evolutiva, Museo Nacional de Ciencias Naturales, CSIC, Madrid, Spain.

11- Centro Austral de Investigaciones Científicas – Consejo Nacional de Investigaciones Científicas y Técnicas (CADIC-CONICET), Ushuaia, Argentina.

12- Instituto de Ciencias Polares, Ambiente y Recursos Naturales, Universidad Nacional de Tierra del Fuego, Ushuaia, Argentina.

13- Wildlife Conservation Society, Buenos Aires, Argentina.

14- PPG in Vertebrate Biology, Pontificia Universidade Católica de Minas Gerais, Belo Horizonte, Brazil.

15- Museum of Vertebrate Zoology and Department of Integrative Biology, 3101 Valley Life Science Building, University of California, Berkeley, CA 94720, USA.

16- Fondo de Desarrollo de Áreas Prioritarias (FONDAP), Center for Genome Regulation (CRG), Santiago, Chile.

Corresponding Author: Juliana A. Vianna, Pontificia Universidad Católica de Chile, Departamento de Ecosistemas y Medio Ambiente, Vicuña Mackenna 4860, Macul, Santiago, Chile, phone number: 56-223547210, jvianna@uc.cl

**Figures Supplementary Material**


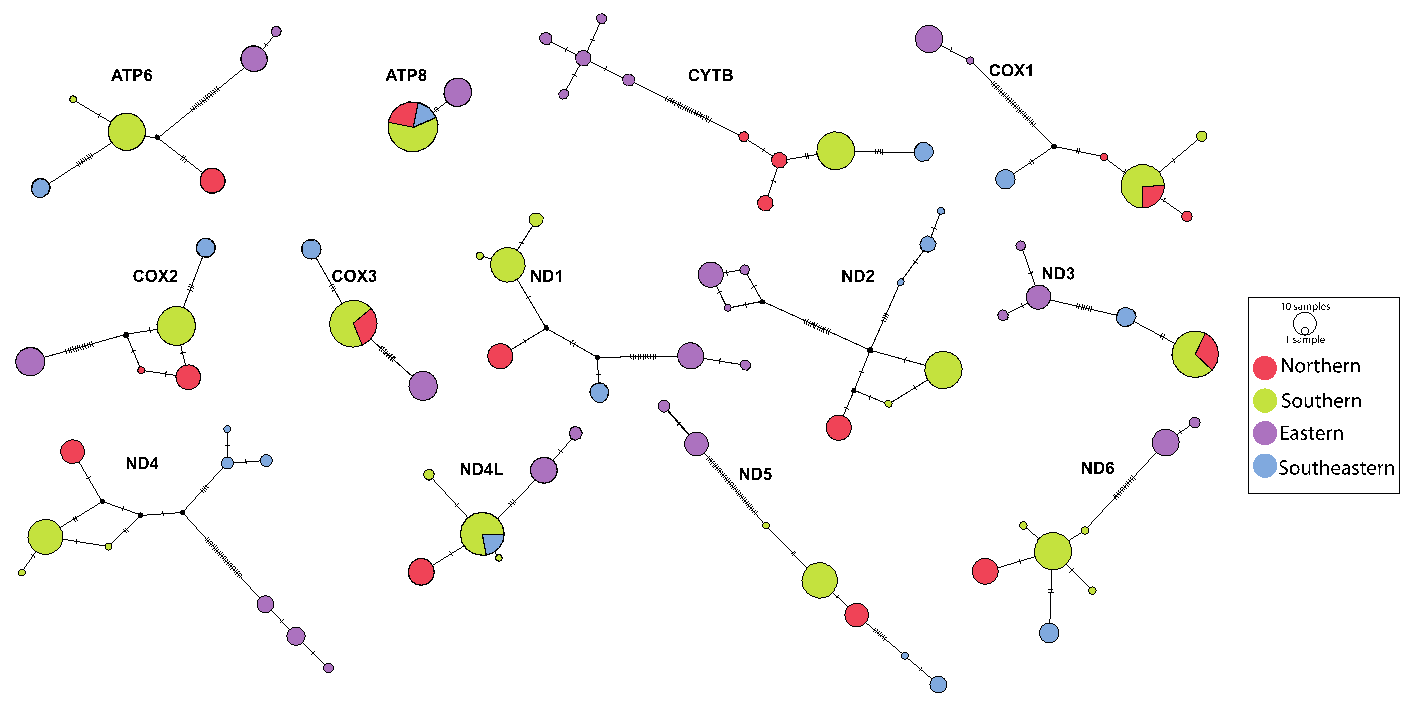


Figure S1: Median Joining Network of the 13 mitochondrial PCG to evaluate genealogical relationship among haplotypes.

**
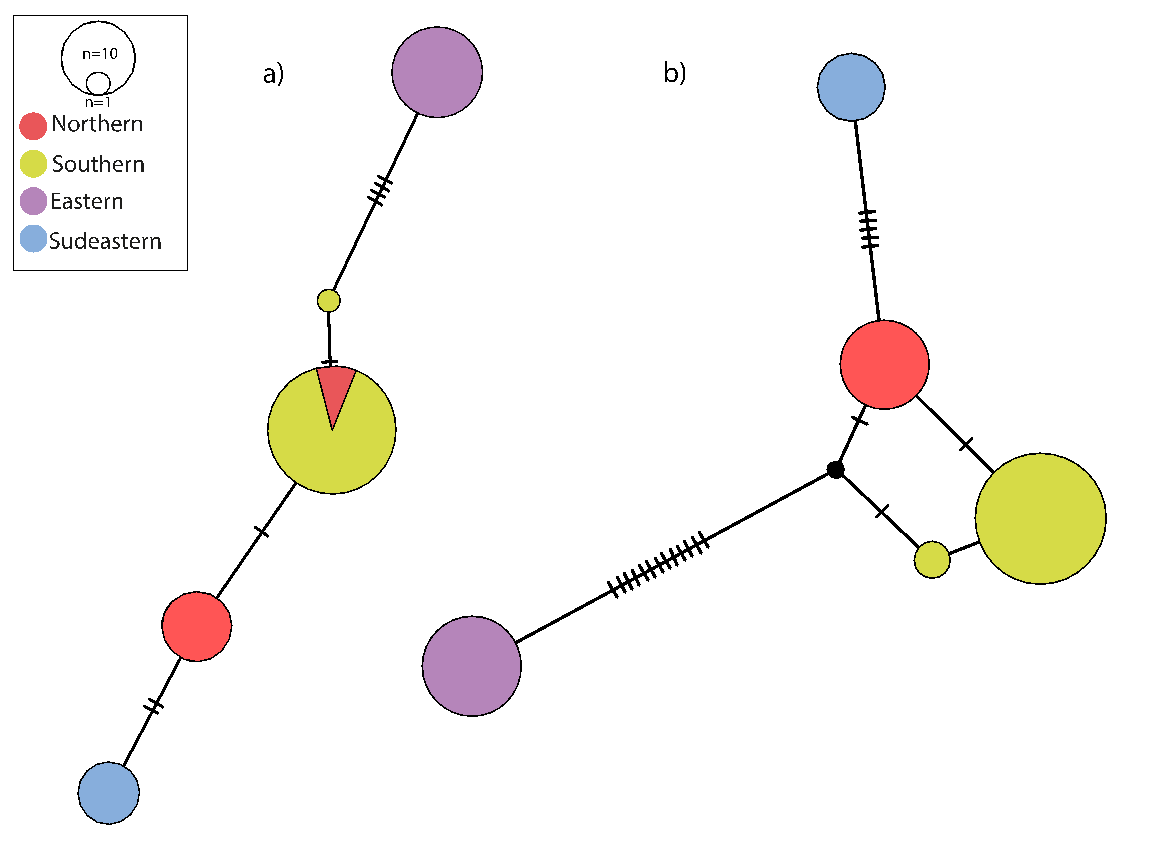
**

Figure S2: Genealogic relationship among haplotypes of ribosomal genes a)12S and b)16S.

Figure S3: Genetic distance between pairs of mitogenomes using Maximum Composite Likelihood.

**Tables supplementary Material**

Table S1: Sampling locations and environmental information extract from Pertierra et al, 2020). SST: Sea Surface Temperature, PP: Primary productivity.

| **Gentoo penguin Locations** | **Latitude** | **Longitude** | **Environmental information** | | | | | | | | |
| --- | --- | --- | --- | --- | --- | --- | --- | --- | --- | --- | --- |
|  |  |  | **SST** | | | **PP** | | | **Salinity** | | **Ice cover** |
|  |  |  | **Max (o13)** | **Min (o14)** | **Max**  **(o9)** | | **Min (o10)** | **Max (o11)** | | **Min (o12)** | **Max (o1)** |
| Crozet Is. | 46°25'47.30''S | 50°24'16.83''E | 8.26 | 3.56 | 0.005987 | | 0.001014 | 33.94 | | 33.55 | 0 |
| Marion Is. | 46°58'25.81"S | 37°41'54.43"E | 7.85 | 3.92 | 0.005828 | | 0.00174 | 33.99 | | 33.49 | 0 |
| Kerguelen Is. | 49°16'52.28''S | 70°32'27.90''E | 6.21 | 1.66 | 0.019921 | | 0.003048 | 33.98 | | 33.64 | 0 |
| Falkland Is. | 52°20'16.7''S | 59°21'48.02''W | 10.70 | 4.26 | 0.056462 | | 0.007465 | 34.17 | | 33.73 | 0 |
| Martillo Is. | 54°54'1.79"S | 67°23'45.35"W | 9.41 | 4.74 | 0.097493 | | 0.006259 | 34.09 | | 33.64 | 0 |
| Signy Is. | 60°43'54.14"S | 45°35'58.70"W | 1.17 | -1.93 | 0.014932 | | 0.000043 | 34.37 | | 32.44 | 0.828 |
| B. O'Higgins Base. | 63°19'14.64''S | 57°53'55.25''W | 0.13 | -1.93 | 0.038683 | | 0.000034 | 34.52 | | 32.01 | 0.812 |
| Stranger Point | 62°14'14.86''S | 58°35'38.26''W | 1.61 | -1.87 | 0.027137 | | 0.000021 | 34.56 | | 33.16 | 0.784 |
| G. G. Videla Base. | 64°49'26.12''S | 62°51'29.04''W | 1.76 | -1.74 | 0.046855 | | 0.000017 | 34.52 | | 31.61 | 0.808 |

Table S2: Mitogenome size and regions with differences on size among lineages.

| Lineage | Locality | Mitogenome Size |
| --- | --- | --- |
| Eastern | Crozet I. | 16,998 |
|  | Marion I. | 16,998 |
| Southeastern | Kerguelen I. | 16,993 |
| Northern | Falkland/Malvinas I. | 16,995 |
|  | Martillo I. | 16,995 |
| Southern | Signy I. | 16,994 |
|  | O'Higgins B. | 16,994 |
|  | Stranger Point | 16,994 |
|  | Gabriel G. Videla B. | 16,994 |

Table S3: Genetic distance among lineages.

|  | p-distance | |
| --- | --- | --- |
|  | Lower | Higher |
| **Within location**  Crozet Is. | 0 | 0.0013 |
| Marion Is. | 0.0001 | 0.0015 |
| Kerguelen Is. | 0 | 0.0010 |
| Falkland/Malvinas Is. | 0.0001 | 0.0005 |
| Signy I. | 0.0001 | 0.0005 |
| O'higgins Base | 0.0001 | 0.0007 |
| Stranger Point | 0.0001 | 0.0006 |
| Gabriel Gonzalez Videla Base | 0 | 0.0006 |
| **Whithin lineage** |  |  |
| Eastern gentoo | 0 | 0.0015 |
| Southeastern gentoo | 0 | 0.0010 |
| Northern gentoo | 0 | 0.0007 |
| Southern gentoo | 0 | 0.0007 |
| **Between lineages** |  |  |
| Eastern/Southeastern gentoo | 0.0223 | 0.0234 |
| Eastern/Northern gentoo | 0.0209 | 0.0218 |
| Eastern/Southern gentoo | 0.0211 | 0.0220 |
| Southeastern/Northern gentoo | 0.0077 | 0.0084 |
| Southeastern/Southern gentoo | 0.0074 | 0.0083 |
| Northern/Southern gentoo | 0.0022 | 0.0029 |

Table S4: Scheme of partitions and the substitution models

| **Subset** | **Best Model** | **# sites** | **Partition names** |
| --- | --- | --- | --- |
| 1 | HKY+I | 225 | ATP6_pos1 |
| 2 | HKY | 1263 | COB_pos2, NAD4L_pos2, NAD4_pos2, ATP6_pos2, NAD3_pos2 |
| 3 | GTR+I | 898 | COX2_pos3, ATP6_pos3, NAD4_pos3 |
| 4 | K81UF+I | 1440 | ATP8_pos1, NAD4_pos1, NAD2_pos1, NAD5_pos1 |
| 5 | TRN+I | 988 | ATP8_pos2, NAD2_pos2, NAD5_pos2 |
| 6 | TRN | 877 | ATP8_pos3, NAD1_pos3, COX1_pos3 |
| 7 | K81UF+G | 907 | NAD4L_pos1, NAD3_pos1, NAD1_pos1, COB_pos1 |
| 8 | TRN | 489 | NAD3_pos3, COB_pos3 |
| 9 | HKY+I | 728 | COX1_pos1, COX2_pos1 |
| 10 | HKY | 1300 | COX2_pos2, NAD1_pos2, COX1_pos2, COX3_pos2 |
| 11 | TRNEF | 252 | COX3_pos1 |
| 12 | GTR+I | 1287 | NAD2_pos3, NAD4L_pos3, NAD5_pos3, COX3_pos3 |
| 13 | HKY+I | 172 | NAD6_pos1 |
| 14 | HKY | 172 | NAD6_pos2 |
| 15 | GTR | 172 | NAD6_pos3 |

Table S5: Codons under purifying selection detected by FUBAR. N: Number of codons in the sequence, PS: codons under purifying selection, p: proportion of codons under purifying selection.

|  | N | PS | p |
| --- | --- | --- | --- |
| ATP6 | 227 | 14 | 0.06 |
| COX1 | 511 | 28 | 0.05 |
| COX2 | 225 | 9 | 0.04 |
| COX3 | 261 | 9 | 0.03 |
| CYTB | 379 | 17 | 0.04 |
| ND1 | 323 | 9 | 0.03 |
| ND2 | 343 | 9 | 0.03 |
| ND3 | 116 | 5 | 0.04 |
| ND4 | 456 | 16 | 0.04 |
| ND4L | 98 | 4 | 0.04 |
| ND5 | 601 | 24 | 0.04 |
| ND6 | 172 | 8 | 0.05 |

Table S6: Parameters estimates for *codeml* site-model. N: (M0: one-ratio; M1: Nearly neutral; M2: Positive selection, M7: beta; M8: beta & 𝟂 >1)

|  | M0 | M1 | | | | M2a | | | | | |
| --- | --- | --- | --- | --- | --- | --- | --- | --- | --- | --- | --- |
|  | 𝟂 | p0 | p1 | 𝟂0 | 𝟂1 | p0 | p1 | p2 | 𝟂0 | 𝟂1 | 𝟂2 |
| ATP6 | 0.038 | 0.951 | 0.049 | 0 | 1 | 0.955 | 0 | 0.045 | 0 | 1 | 1.137 |
| CYTB | 0.1 | 0.99999 | 0.00001 | 0.100 | 1 | 1 | 0 | 0 | 0.100 | 1 | 4.486 |
| COX1 | 0.019 | 0.99999 | 0.00001 | 0.019 | 1 | 1 | 0 | 0 | 0.019 | 1 | 1 |
| COX2 | 0.021 | 0.99999 | 0.00001 | 0.021 | 1 | 1 | 0 | 0 | 0.021 | 1 | 10.614 |
| COX3 | 0.021 | 0.99999 | 0.00001 | 0.021 | 1 | 1 | 0 | 0 | 0.021 | 1 | 1 |
| ND1 | 0.075 | 0.99999 | 0.00001 | 0.075 | 1 | 0.99996 | 0 | 0.00004 | 0.075 | 1 | 1 |
| ND2 | 0.179 | 0.850 | 0.150 | 0 | 1 | 0.993 | 0 | 0.007 | 0.105 | 1 | 344.382 |
| ND3 | 0.142 | 0.99999 | 0.00001 | 0.142 | 1 | 1 | 0 | 0 | 0.142 | 1 | 25.079 |
| ND4 | 0.110 | 0.884 | 0.116 | 0 | 1 | 0.917 | 0 | 0.083 | 0 | 1 | 1.554 |
| ND4L | 0.248 | 0.99999 | 0.00001 | 0.248 | 1 | 1 | 0 | 0 | 0.248 | 1 | 1 |
| ND5 | 0.097 | 0.902 | 0.098 | 0 | 1 | 0.912 | 0.083 | 0.005 | 0 | 1 | 22.222 |
| ND6 | 0.048 | 0.99999 | 0.00001 | 0.048 | 1 | 1 | 0 | 0 | 0.048 | 1 | 6.975 |
|  |  |  |  |  |  |  |  |  |  |  |  |
|  | M7 | | M8 | | | | |  |  |  |  |
|  | p | q | p0 | p1 | p | q | 𝟂 |  |  |  |  |
| ATP6 | 0.009 | 0.172 | 0.955 | 0.045 | 0.005 | 7.951 | 1.137 |  |  |  |  |
| CYTB | 11.084 | 99 | 0.99999 | 0.00001 | 8.954 | 73.086 | 1 |  |  |  |  |
| COX1 | 1.928 | 99 | 0.99999 | 0.00001 | 1.927 | 99 | 1 |  |  |  |  |
| COX2 | 2.164 | 99 | 0.99999 | 0.00001 | 2.164 | 99 | 1 |  |  |  |  |
| COX3 | 0.035 | 1.103 | 0.99999 | 0.00001 | 2.149 | 99 | 1 |  |  |  |  |
| ND1 | 8.111 | 99 | 0.970 | 0.030 | 0.005 | 0.096 | 1 |  |  |  |  |
| ND2 | 0.005 | 0.028 | 0.99344 | 0.00656 | 11.722 | 99 | 344.789 |  |  |  |  |
| ND3 | 16.472 | 99 | 0.99999 | 0.00001 | 16.474 | 99 | 15.573 |  |  |  |  |
| ND4 | 11.864 | 96.835 | 0.918 | 0.083 | 0.005 | 0.155 | 1.535 |  |  |  |  |
| ND4L | 14.639 | 44.122 | 0.99999 | 0.00001 | 32.700 | 99 | 1 |  |  |  |  |
| ND5 | 10.188 | 95.832 | 0.994 | 0.006 | 0.006 | 0.103 | 16.307 |  |  |  |  |
| ND6 | 5.071 | 99 | 0.998 | 0.002 | 0.008 | 0.140 | 1 |  |  |  |  |

Table S7: Parameters estimates in branch-site model A, where p indicates the proportion of sites that belong to each category.

|  |  | Purifying s. | | | Neutral evolution | | | Positive s. + purif. back | | | Positive s. + neutral back | | |
| --- | --- | --- | --- | --- | --- | --- | --- | --- | --- | --- | --- | --- | --- |
|  |  | p0 | 𝟂Back | 𝟂foreg | p1 | 𝟂Back | 𝟂foreg | p2a | 𝟂Back | 𝟂foreg | p2b | 𝟂Back | 𝟂foreg |
| ATP6 | E | 0.57 | 0 | 0 | 0.03 | 1 | 1 | 0.38 | 0 | 4.28 | 0.02 | 1 | 4.28 |
|  | S | 1 | 0.04 | 0.04 | 0 | 1 | 1 | 0 | 0.04 | 2.91 | 0 | 1 | 2.91 |
|  | SE | 0.66 | 0 | 0 | 0.03 | 1 | 1 | 0.29 | 0 | 3.96 | 0.01 | 1 | 3.96 |
|  | N | 1 | 0.04 | 0.04 | 0 | 1 | 1 | 0 | 0.04 | 2.91 | 0 | 1 | 2.91 |
|  | N+S | 1 | 0.04 | 0.04 | 0 | 1 | 1 | 0 | 0.04 | 2.90 | 0 | 1 | 2.90 |
|  | N+S+SE * | 0 | 0.02 | 0.02 | 0 | 1 | 1 | **1** | **0.02** | **999** | 0 | 1 | 999 |
| COX1 | E | 1 | 0.02 | 0.02 | 0 | 1 | 1 | 0 | 0.02 | 2.91 | 0 | 1 | 2.91 |
|  | S | 1 | 0.02 | 0.02 | 0 | 1 | 1 | 0 | 0.02 | 2.90 | 0 | 1 | 2.90 |
|  | SE | 1 | 0.02 | 0.02 | 0 | 1 | 1 | 0 | 0.02 | 2.91 | 0 | 1 | 2.91 |
|  | N | 1 | 0.02 | 0.02 | 0 | 1 | 1 | 0 | 0.02 | 2.90 | 0 | 1 | 2.90 |
|  | N+S | 1 | 0.02 | 0.02 | 0 | 1 | 1 | 0 | 0.02 | 2.90 | 0 | 1 | 2.90 |
|  | N+S+SE | 1 | 0.02 | 0.02 | 0 | 1 | 1 | 0 | 0.02 | 1.00 | 0 | 1 | 1 |
| COX2 | E | 1 | 0.02 | 0.02 | 0 | 1 | 1 | 0 | 0.02 | 2.91 | 0 | 1 | 2.91 |
|  | S | 1 | 0.02 | 0.02 | 0 | 1 | 1 | 0 | 0.02 | 2.92 | 0 | 1 | 2.92 |
|  | SE | 1 | 0.02 | 0.02 | 0 | 1 | 1 | 0 | 0.02 | 2.91 | 0 | 1 | 2.91 |
|  | N | 1 | 0.02 | 0.02 | 0 | 1 | 1 | 0 | 0.02 | 2.92 | 0 | 1 | 2.92 |
|  | N+S | 1 | 0.02 | 0.02 | 0 | 1 | 1 | 0 | 0.02 | 2.90 | 0 | 1 | 2.90 |
|  | N+S+SE * | 0 | 0.01 | 0.01 | 0 | 1 | 1 | **1** | **0.01** | **999** | 0 | 1 | 999 |
| COX3 | E | 1 | 0.02 | 0.02 | 0 | 1 | 1 | 0 | 0.02 | 2.93 | 0 | 1 | 2.93 |
|  | S | 1 | 0.02 | 0.02 | 0 | 1 | 1 | 0 | 0.02 | 2.90 | 0 | 1 | 2.90 |
|  | SE | 1 | 0.02 | 0.02 | 0 | 1 | 1 | 0 | 0.02 | 2.92 | 0 | 1 | 2.92 |
|  | N | 1 | 0.02 | 0.02 | 0 | 1 | 1 | 0 | 0.02 | 2.90 | 0 | 1 | 2.90 |
|  | N+S | 1 | 0.02 | 0.02 | 0 | 1 | 1 | 0 | 0.02 | 2.89 | 0 | 1 | 2.89 |
|  | N+S+SE | 1 | 0.02 | 0.02 | 0 | 1 | 1 | 0 | 0.02 | 2.67 | 0 | 1 | 2.67 |
| CYTB | E | 1 | 0.09 | 0.09 | 0 | 1 | 1 | 0 | 1 | 2.93 | 0 | 1 | 2.93 |
|  | S | 1 | 0.09 | 0.09 | 0 | 1 | 1 | 0 | 0.09 | 2.92 | 0 | 1 | 2.92 |
|  | SE | 1 | 0.09 | 0.09 | 0 | 1 | 1 | 0 | 0.09 | 2.93 | 0 | 1 | 2.93 |
|  | N | 1 | 0.09 | 0.09 | 0 | 1 | 1 | 0 | 0.09 | 1 | 0 | 1 | 1 |
|  | N+S | 1 | 0.09 | 0.09 | 0 | 1 | 1 | 0 | 0.09 | 2.92 | 0 | 1 | 2.92 |
|  | N+S+SE | 1 | 0.09 | 0.09 | 0 | 1 | 1 | 0 | 0.09 | 1 | 0 | 1 | 1 |
| ND1 | E | 1 | 0.07 | 0.07 | 0 | 1 | 1 | 0 | 0.07 | 2.91 | 0 | 1 | 2.91 |
|  | S | 1 | 0.07 | 0.07 | 0 | 1 | 1 | 0 | 0.07 | 1 | 0 | 1 | 1 |
|  | SE | 1 | 0.07 | 0.07 | 0 | 1 | 1 | 0 | 0.07 | 2.94 | 0 | 1 | 2.94 |
|  | N | 1 | 0.07 | 0.07 | 0 | 1 | 1 | 0 | 0.07 | 2.91 | 0 | 1 | 2.91 |
|  | N+S | 1 | 0.07 | 0.07 | 0 | 1 | 1 | 0 | 0.07 | 1 | 0 | 1 | 1 |
|  | N+S+SE | 0.99 | 0.04 | 0.04 | 0 | 1 | 1 | 0.01 | 0.04 | 999 | 0 | 1 | 999 |
| ND2 | E | 0.72 | 0 | 0 | 0.11 | 1 | 1 | 0.14 | 0 | 2.96 | 0.02 | 1 | 2.96 |
|  | S | 0.73 | 0 | 0 | 0.12 | 1 | 1 | 0.14 | 0 | 2.92 | 0.02 | 1 | 2.92 |
|  | SE | 0.69 | 0 | 0 | 0.11 | 1 | 1 | 0.17 | 0 | 2.89 | 0.03 | 1 | 2.89 |
|  | N | 0.73 | 0 | 0 | 0.12 | 1 | 1 | 0.13 | 0 | 2.95 | 0.02 | 1 | 2.95 |
|  | N+S | 0.86 | 0 | 0 | 0.14 | 1 | 1 | 0 | 0 | 1 | 0 | 1 | 1 |
|  | N+S+SE | 0.73 | 0 | 0 | 0.12 | 1 | 1 | 0.13 | 0 | 2.90 | 0.02 | 1 | 2.90 |
| ND3 | E | 1 | 0.12 | 0.12 | 0 | 1 | 1 | 0 | 0.12 | 2.91 | 0 | 1 | 2.91 |
|  | S | 1 | 0.12 | 0.12 | 0 | 1 | 1 | 0 | 0.12 | 2.91 | 0 | 1 | 2.91 |
|  | SE | 1 | 0.12 | 0.12 | 0 | 1 | 1 | 0 | 0.12 | 2.91 | 0 | 1 | 2.91 |
|  | N | 0.99999 | 0.12 | 0.12 | 0 | 1 | 1 | 0 | 0.12 | 2.91 | 0 | 1 | 2.91 |
|  | N+S | 0.99999 | 0.12 | 0.12 | 0 | 1 | 1 | 0 | 0.12 | 2.91 | 0 | 1 | 2.91 |
|  | N+S+SE | 0.99 | 0.10 | 0.10 | 0 | 1 | 1 | 0.01 | 0.10 | 161 | 0 | 1 | 161 |
| ND4 | E | 0.90 | 0 | 0 | 0.10 | 1 | 1 | 0 | 0 | 1 | 0 | 1 | 1 |
|  | S | 0.09 | 0 | 0 | 0.01 | 1 | 1 | 0.81 | 0 | 6.33 | 0.09 | 1 | 6.33 |
|  | SE | 0.89 | 0.09 | 0.09 | 0.02 | 1 | 1 | 0.09 | 0.09 | 2.89 | 0.00 | 1 | 2.89 |
|  | N | 0.00 | 0 | 0 | 0.00 | 1 | 1 | 0.90 | 0 | 8.95 | 0.10 | 1 | 8.95 |
|  | N+S | 0.69 | 0 | 0 | 0.07 | 1 | 1 | 0.22 | 0 | 1 | 0.02 | 1 | 1 |
|  | N+S+SE | 0.90 | 0 | 0 | 0.10 | 1 | 1 | 0 | 0 | 1 | 0 | 1 | 1 |
| ND4L | E | 1 | 0.22 | 0.22 | 0 | 1 | 1 | 0 | 0.22 | 2.90 | 0 | 1 | 2.90 |
|  | S | 1 | 0.22 | 0.22 | 0 | 1 | 1 | 0 | 0.22 | 2.91 | 0 | 1 | 2.91 |
|  | SE | 1 | 0.22 | 0.22 | 0 | 1 | 1 | 0 | 0.22 | 2.58 | 0 | 1 | 2.58 |
|  | N | 1 | 0.22 | 0.22 | 0 | 1 | 1 | 0 | 0.22 | 2.88 | 0 | 1 | 2.88 |
|  | N+S | 1 | 0.22 | 0.22 | 0 | 1 | 1 | 0 | 0.22 | 2.92 | 0 | 1 | 2.92 |
|  | N+S+SE | 1 | 0.22 | 0.22 | 0 | 1 | 1 | 0 | 0.22 | 2.90 | 0 | 1 | 2.90 |
| ND5 | E | 0.79 | 0 | 0 | 0.08 | 1 | 1 | 0.13 | 0 | 2.94 | 0.01 | 1 | 2.94 |
|  | S | 1.00 | 0.09 | 0.09 | 0 | 1 | 1 | 0.00 | 0.09 | 2.89 | 0 | 1 | 2.89 |
|  | SE * | 0.79 | 0 | 0 | 0.08 | 1 | 1 | **0.12** | **0** | **2.93** | 0.01 | 1 | 2.93 |
|  | N | 0.00 | 0 | 0 | 0.00 | 1 | 1 | 0.91 | 0 | 8.78 | 0.09 | 1 | 8.78 |
|  | N+S * | 0 | 0 | 0 | 0 | 1 | 1 | **0.92** | **0** | **50.45** | 0.08 | 1 | 50.45 |
|  | N+S+SE | 0.91 | 0 | 0 | 0.09 | 1 | 1 | 0.00 | 0 | 92.94 | 0 | 1 | 92.94 |
| ND6 | E | 1 | 0.04 | 0.04 | 0 | 1 | 1 | 0 | 0.04 | 2.78 | 0 | 1 | 2.78 |
|  | S | 1 | 0.04 | 0.04 | 0 | 1 | 1 | 0 | 0.04 | 2.88 | 0 | 1 | 2.88 |
|  | SE | 1 | 0.04 | 0.04 | 0 | 1 | 1 | 0 | 0.04 | 2.93 | 0 | 1 | 2.93 |
|  | N | 1 | 0.04 | 0.04 | 0 | 1 | 1 | 0 | 0.04 | 2.91 | 0 | 1 | 2.91 |
|  | N+S | 1 | 0.04 | 0.04 | 0 | 1 | 1 | 0 | 0.04 | 2.90 | 0 | 1 | 2.90 |
|  | N+S+SE | 1 | 0.04 | 0.04 | 0 | 1 | 1 | 0 | 0.04 | 2.88 | 0 | 1 | 2.88 |
